# Desorption of Water from Distinct Step Types on a Curved Silver Crystal

**DOI:** 10.3390/molecules190810845

**Published:** 2014-07-25

**Authors:** Jakrapan Janlamool, Dima Bashlakov, Otto Berg, Piyasan Praserthdam, Bunjerd Jongsomjit, Ludo B. F. Juurlink

**Affiliations:** 1 Center of Excellence on Catalysis and Catalytic Reaction Engineering, Department of Chemical Engineering, Faculty of Engineering, Chulalongkorn University, Bangkok 10330, Thailand; 2 Leiden Institute of Chemistry, Leiden University, PO BOX 9502, 2300 RA Leiden, The Netherlands

**Keywords:** silver, water, adsorption, single crystal, steps, surface science

## Abstract

We have investigated the adsorption of H_2_O onto the A and B type steps on an Ag single crystal by temperature programmed desorption. For this study, we have used a curved crystal exposing a continuous range of surface structures ranging from [5(111) × (100)] via (111) to [5(111) × (110)]. LEED and STM studies verify that the curvature of our sample results predominantly from monoatomic steps. The sample thus provides a continuous array of step densities for both step types. Desorption probed by spatially-resolved TPD of multilayers of H_2_O shows no dependence on the exact substrate structure and thus confirms the absence of thermal gradients during temperature ramps. In the submonolayer regime, we observe a small and linear dependence of the desorption temperature on the A and B step density. We argue that such small differences are only observable by means of a single curved crystal, which thus establishes new experimental benchmarks for theoretical calculation of chemically accurate binding energies. We propose an origin of the observed behavior based on a “two state” desorption model.

## 1. Introduction

The kinetics of many heterogeneously catalyzed reactions depend strongly on the structure and size of transition metal particles [1]. In recent years, theoretical studies have begun to unravel the origin of such structure sensitivity [2,3]. Particle size dependence often stems from the occurrence of particular arrangements of surface atoms. These clusters critically lower the activation barrier for dissociation of a small molecule or the assembly of an intermediate from surface-bound species. Amongst others, ammonia synthesis over Ru and methanol synthesis over Cu/ZnO have been shown to depend on particular configurations of atoms at monoatomic steps occurring at the surface of catalyst particles [4,5].

Besides using grown [6] or deposited (e.g., [7,8]) catalytic particles on metal oxide substrates, fundamental experimental investigations of heterogeneous catalysis often employ flat, well polished, single crystal metal surfaces. On low Miller index surfaces, the coordination number of surface atoms is maximized. To investigate the influence of surface sites with lower coordination, defects can be added either by choosing a high Miller index surface plane, by intentionally sputtering the surface without subsequent annealing, or by adding fresh but incomplete metal layers, e.g., by vapor deposition. On the other hand, it was recognized several decades ago that curved single crystal samples may provide benefits to experimental studies probing chemical and physical phenomena that depend on surface structure [9,10,11,12,13,14,15]. The presence of a range of locally uniform steps and kinks on a single sample circumvents common sources of sample-to-sample heterogeneity, such as different levels of contamination or differences in the accuracy of temperature measurement. Also, it simplifies experimental procedures as the breaking of vacuum or repetitive loading of samples is reduced to an absolute minimum. Nonetheless, the total number of studies employing partially curved or fully cylindrical single crystals in surface science and catalysis remains very low. We expect that this is due, at least in part, to new experimental challenges: limited ability to map the exact surface structure, limited spatial resolution of various surface science probes, metallurgical reconstructions and step bunching which relax local surface energy, difficulties growing large single crystal metal boules, and the lack of polishing techniques that yield a finish comparable to flat single crystal surfaces.

Recently, interest in curved samples for physical and chemical studies probing structure sensitivity has revived [16,17,18]. Using Scanning Tunneling Microscopy (STM) and Angle-Resolved Photoemission Spectroscopy (ARPES), Ortega and co-workers showed that faceting and step bunching were present on a curved Au sample at particular angles close to the (111) plane [19], but not present on similar Ag and Cu samples. The stepped lattice was found to be more unstable for Cu than for Ag [20]. We recently employed a cylindrical Ni single crystal, together with supersonic molecular beam techniques, to map the reactivity dependence toward hydrogen dissociation of various step densities, step and terraces types, and for a wide range of impact energies [16]. Low energy electron diffraction (LEED) studies of our Ni sample suggest that monoatomic steps cover a large part of the circumference of the cylinder [21]. Sykes and Gellman recently began to employ a series of dome shaped Cu single crystals for STM [17,22] and were able to relate surface structure to oxidation kinetics by means of Auger Electron Spectroscopy (AES) [17].

Here, we employ a curved Ag single crystal that was prepared by a new polishing technique to investigate the surface structure dependence of water adsorption/desorption. Details about the structure and preparation of this sample can be found in the experimental section. For hydrophilic metals and low Miller index surfaces, the sizeable literature available on the H_2_O-surface interaction has been reviewed in great detail [23,24,25]. Fewer studies have focused on stepped and hydrophobic surfaces. In recent studies, steps have been shown to exert unexpected influences on water adsorption. For example, we and others have found that water preferentially decorates Pt step sites prior to wetting terraces [26,27]. The most common step types separating (111) terraces, *i.e.*, the A or (100) and B or (110) step types, have now been shown to have different binding energies for H_2_O [28]. They also differ in their tendency to form OH in coadsorption with oxygen [29] and have different behavior in coadsorption with H (or D) atoms [27]. Whereas coadsorption with hydrogen in the presence of the B step type is not very different from the (111) terrace, the A step type induces hydrophobicity and reduced H-D exchange reactivity for fully hydrogenated Pt surfaces. This behavior remains unaltered over a (111) terrace length variation of four to eight atoms [30].

A clear influence of step structure may be expected when there is a small energetic difference between the adsorbate-surface and adsorbate-adsorbate interaction. In these cases, the exact atomic structure of the substrate may just “tip the scale”. For hydrophobic metal surfaces, this influence of steps may be expected to be smaller. Zeroth-order desorption kinetics from Ag(111) and Ag(100) observed by Klaua and Madey led to the conclusion that these surfaces are hydrophobic [31]. They found a desorption enthalpy near the sublimation energy of ice and attributed it to a weakened H_2_O-metal interaction compared with, e.g., Pt [31,32]. As Ag is in general a rather non-reactive metal, potential influences of low-level contamination of oxygen—which may influence water structures significantly [33,34]—are also easily avoided. A study of water adsorption on a curved Ag sample therefore tests our ability to measure small differences in the binding of molecules to different surface structures. In the present study, we first characterize the step density as a function of cylindrical azimuth; we then show that the binding energy difference of (111) terraces interrupted by A *vs.* B step sites is indeed very small, but observable when using a curved single crystal sample. Finally, we consider a possible origin for the small shift in peak desorption temperature observed in spatially-resolved temperature programmed desorption (TPD) studies at submonolayer coverages.

## 2. Results and Discussion

**Figure 1:**
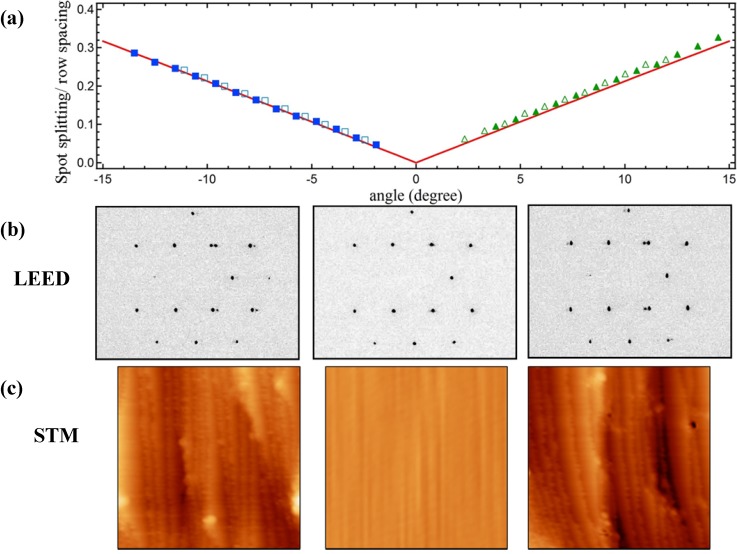
(**a**) Spot-splitting/row-spacing ratio as a function of azimuthal angle; open *vs.* closed symbols represent two data sets collected on different days, red solid lines indicate expected values; (**b**) Images of color-inverted LEED patterns taken at −1.5 mm (−5.7°, left), 0.00 mm (0°, middle), and +1.5 mm (+5.7°, right) from the crystal center; (**c**) STM images (50 × 50 nm^2^) taken at −1.6 mm (−6.1°, left), 0.00 mm (0°, middle), and +1.6 mm (+6.1°, right) from the crystal center.

Figure 1 summarizes results from LEED and STM studies. Figure 1a shows the spot-splitting/row-spacing ratio as a function of azimuthal position from LEED patterns that were recorded while translating the crystal normal to the impacting electron beam. We simultaneously adjust the crystal’s position to maintain a constant LEED-to-sample distance. Figure 1b shows representative LEED patterns. As explained by Henzler [35], diffraction from the stepped structure peaks at regularly spaced angles, ∆*φ*, depending only on the terrace width (Na + g) and the step height (d) (see also graphical illustration in the Experimental section:

∆*φ* = λ/[(Na + g)cos*φ* − dsin*φ*] (1)

For the same number of terrace atoms, N, the spot-splitting/row-spacing ratio for the A type step (at negative angles) is slightly smaller than for the B type step (at positive angles). The difference is a consequence of the different value of the horizontal offset (g) between the exposed Ag lattice of successive terraces. The ratio has been calculated for a large number of stepped structures with integer N terrace atoms by van Hove and Somorjai, who also suggested the [N(terrace type) × (step type)] nomenclature [36]. When expressed in terms of angular position on a cylindrical crystal, their tabulated values (for structures that are exposed on our Ag crystal) are a linear function of azimuthal angle. In Figure 1a we plot this function (solid red lines) as described in our studies of a cylindrical Ni single crystal [21] together with two data sets of the experimentally determined ratio (solid and open symbols). The coincidence of the experimental data with predictions of the Henzler model indicates that our curved surface yields the expected average local step density at any position away from the (111) center. Furthermore, as explained in detail in ref [21], one can also verify that steps are truly monoatomic by determining the electron energies at which the (0,0) beam shows singlets and doublets. We have performed this analysis at various azimuthal positions and find that the curvature of the crystal can only be explained by the predominance of monoatomic steps. The same conclusion was drawn by Ortega and coworkers for their very similar curved Ag single crystal [18].

Our STM data, taken later in the UHV-STM apparatus, corroborate these findings. Typical images of ~50 × 50 nm^2^ are shown in Figure 1c. The middle image shows a large and flat (111) area found near the middle of the crystal. The other images show large areas dominated by monoatomic steps. Protrusions appearing as white and black spots cover <2% of the surface area. They have an apparent height on the order of 1 nm and remain unidentified, as we detect no elements other than Ag in AES spectra. These protrusions could not be removed by extensive sputtering-annealing or oxidation-reduction cycles, which suggests that they are chemically inert remnants of the polishing process. They are uniformly scattered across the crystal. Although they may influence adsorption/desorption of molecules located in their vicinity, they cannot explain trends as described below for water desorption.

**Figure 2:**
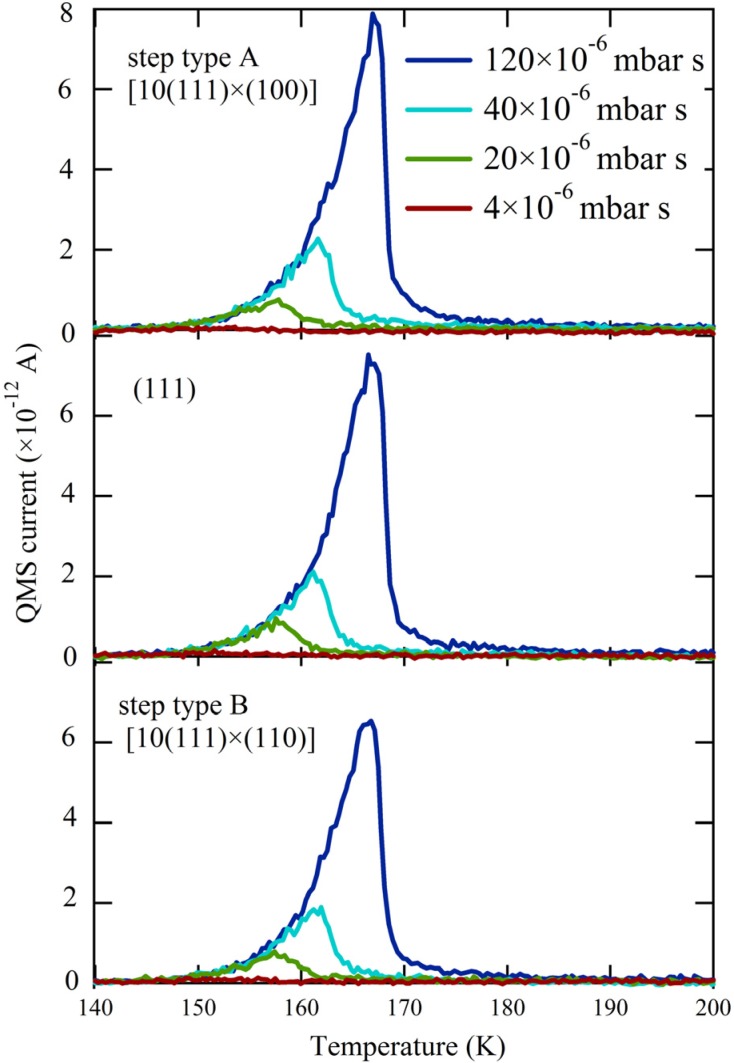
TPD spectra obtained at three separate locations—[10(111) × (100)](top), (111) (middle), and [10(111) × (110)](bottom)—for increasing exposures to H_2_O (colored traces, expressed in relative dose of the H_2_O/He mixture).

Figure 2 shows three series of H_2_O TPD spectra obtained with the QMS canister at three different positions over the crystal. The surface structure centered at the QMS opening is indicated. For each series, we changed both exposure time and dosing pressure of the H_2_O/He mixture to vary the total exposure 30-fold. The results for the (111) plane (middle panel) are consistent with previous studies [25,31,37,38]. A single desorption feature is observed at all coverages, with overlapping leading edges. This behavior is typical for zeroth-order desorption kinetics and is often interpreted to indicate water desorption from a hydrophobic surface. It is not possible to determine the absolute amount of desorbing water from the integrated QMS response without a separate reference. Therefore we recorded the QMS response for a monolayer of water desorbing from Pt(533) [27] under the same experimental conditions (*i.e.*, crystal-to-QMS aperture distance and QMS settings). On these grounds, the largest traces shown in Figure 2 correspond to the desorption of 2 ML (monolayers).

**Figure 3:**
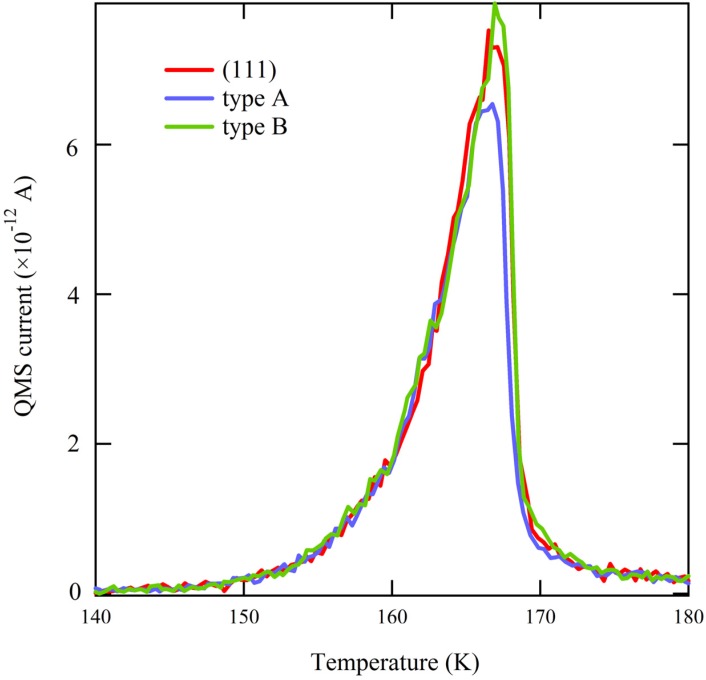
Comparison of the onset of desorption of ~2 ML H_2_O from highly stepped surfaces (types A and B) and flat Ag(111).

Apparently, water desorption in the near-monolayer regime is not affected by the presence of A or B type steps; all traces in Figure 2 from comparable doses are identical. When comparing the onsets of multilayered water desorption for identical amounts of adsorbed H_2_O (Figure 3), the traces overlap perfectly. Since water desorption in the multilayer regime is independent of the underlying metal surface structure, this result allows us to conclude that there is no measurable temperature gradient present during the TPD ramp across the curved surface. This conclusion is crucial when we focus on desorption of very small quantities of water.

**Figure 4:**
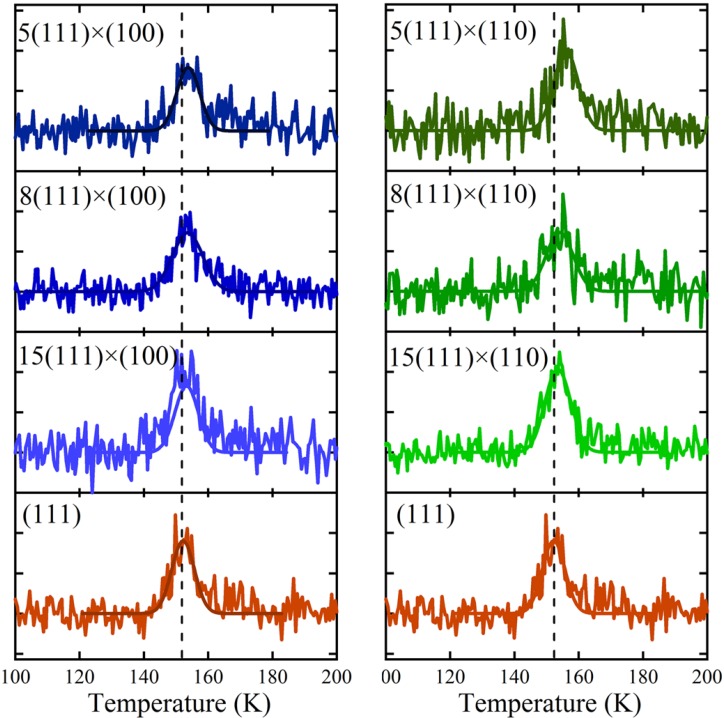
Series of spatially-resolved TPD spectra for 0.06–0.08 ML exposures to H_2_O for the (left) A step type [N(111) × (100)] and (right) B step type [N(111) × (110)]. Solid lines are fits to the data using Gaussian functions.

Thermal desorption spectra for 0.06–0.08 ML H_2_O are shown in Figure 4 for (111) (left and right bottom traces) and various average terrace widths separated by A (left) and B (right) type steps. A small shift in the peak desorption temperature as a function of step density is apparent when comparing TPD traces for each type of step. To facilitate comparison, the temperature of maximum flux from Ag(111) is indicated. We fit each desorption profile using a single Gaussian function and determine the peak’s amplitude (A), width (∆T), and peak desorption temperature (T_p_). The fits are shown in Figure 4 as solid traces through the data.

**Figure 5:**
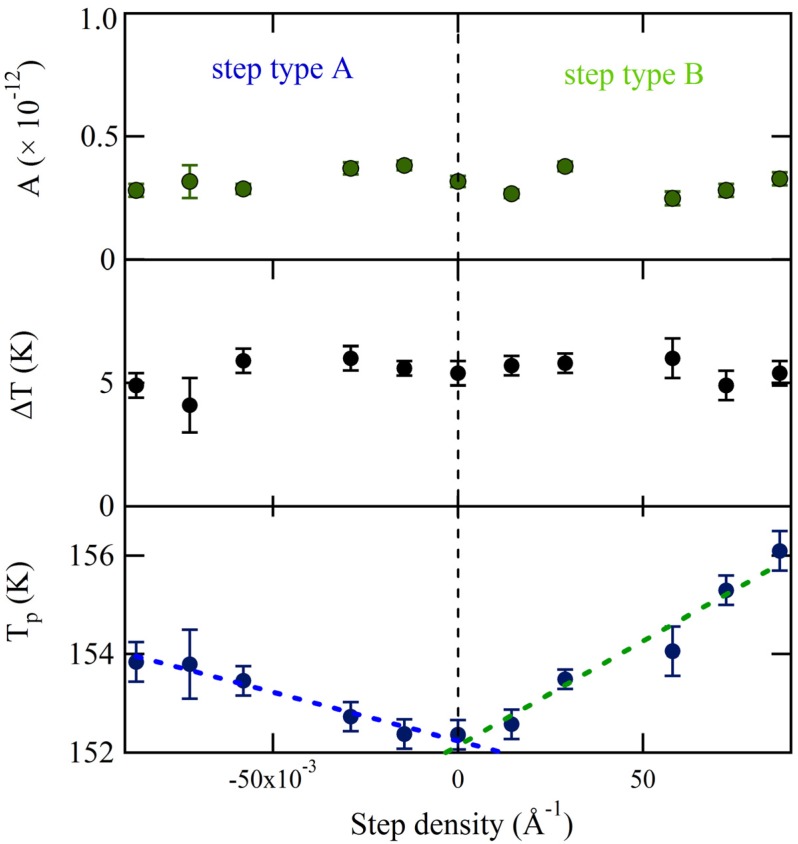
Amplitude (A), peak width (∆T) and peak temperature (T_p_) determined from fitting spatially-resolved TPD spectra with Gaussian functions.

The values of the three fitting parameters are plotted *vs.* step density in Figure 5. The uncertainties as determined by the fitting procedure are shown as error bars. The desorption amplitude (A) and width (∆T) are nearly constant. No clear trend with step density is observed. The peak desorption temperature (T_p_), however, depends significantly on step density over the entire range for both A and B. Translating the crystal over +/–3 mm results in a shift of ~1.5 K for the A step type and ~3.5 K for the B step type. Over this range, the central surface structure from which desorption is probed changes from approximately [6(111) × (100)] to [6(111) × (110)]. The temperature shift appears to be proportional to step density. Linear fits are shown as dashed lines. These individual fits suggest that T_p_ for the “infinite” (111) plane is 152.2 ± 0.1 K and 152.1 ± 0.2 K, as determined from the A and B steps respectively. The slopes are 19.7 ± 1.8 K × Å and 42.4 ± 4.4 K × Å. Considering the uncertainty of the best-fit parameters and the negligible temperature gradient across the crystal as determined from Figure 3, we conclude that the A and B type steps influence water desorption in an experimentally measurable and different manner. The B step type induces a peak temperature shift approximately twice as large as the A step type. We have attempted to relate this shift to the heat of adsorption from terraces and steps using various standard TPD analyses. However, the sensitivity of our data is limited by two experimental necessities: low H_2_O coverage (to probe steps only) and narrow QMS aperture (to ensure maximal spatial resolution); unfortunately, the present signal-to-noise ratio does not allow us to extract reliable adsorption-enthalpy values.

The results presented here are noteworthy for two reasons. First, the peak temperature shift is small but significant. To our knowledge, this is the first time that a characteristic change in TPD features of this magnitude has been attributed to surface structure. The difference is so small that it would be difficult to determine using multiple flat single crystals. At least 2 or 3 samples with widely varying step densities, of both step types, and an Ag(111) surface would be required to establish this trend. Even then, random sample-to-sample temperature measurement error (caused by irreproducible mounting of samples and thermocouples) is likely to dominate the intrinsically lower random error of a single sample measured multiple times, as here. This point also warns against comparison of results obtained using various (flat or curved) samples when studying adsorption/desorption behavior for systems that may be very sensitive to defect concentrations, or show only small changes with substrate structure or co-adsorbate coverage. Second, considering the shape and near invariance in peak width, it is not immediately obvious how a linear dependence of a peak desorption temperature on step density for adsorbates in the submonolayer regime is to be interpreted. To our knowledge, such a phenomenon has also not been reported before.

To guide our consideration of possible origins for the observed desorption temperature dependence, we first summarize the results of previous publications on the adsorption of water to Ag(111) and related surfaces. The first report of water desorption from Ag(111) by Klaua and Madey [31] shows a single peak with a desorption maximum shifting from 175 K to higher temperatures with increased dosing. In line with zeroth-order desorption kinetics, the leading edges of all traces overlap. Close inspection of their TPD data shows the onset of desorption at ~141 K. This is in excellent agreement with our results for Ag(111) at very low water coverages, as shown in Figure 4. The same analysis for their desorption data from Ag(100) suggests an onset near 145 K. The first desorption data from Ag(110) by Stuve, Madix and Sexton [39] unfortunately shows a non-flat baseline prior to the desorption, hence these data do not allow us to extract the onset temperature. In a later publication, the onset appears ~140 K for 0.06 ML of water [38]. None of these surfaces showed any evidence for long-range ordering of water molecules by LEED or Electron Stimulated Desorption in Ion Angular Distributions (ESDIAD). The desorption features were interpreted to indicate either dissolving of water clusters in the submonolayer regime and/or sublimation from three-dimensional crystallites. For all these substrates, the intermolecular forces were apparently greater than the interaction of water with the Ag substrate. Multilayered features simply form as a consequence of an increased chance for water molecules to impinge onto two-dimensional ice clusters with increasing exposure and the absence of a dominant driving force to wet the substrate.

In a more recent series of experiments that imaged water on Ag(111) using low temperature STM in the submonolayer regime, Morgenstern and coworkers found single protrusions for water dosed at 70 K [40,41]. These protrusions were interpreted as cyclic water hexamers. Larger stable clusters consisting of heptamers, octamers, and nonamers are also observed when water is dosed at 17 K [42]. The hexamers are buckled, with alternating H-bond lengths as a consequence of a competition between H_2_O’s simultaneous tendency to bond with the substrate and act as a hydrogen bond acceptor. At the higher dosing temperature, hexamers mostly conglomerate in large water-covered patches without long-range order [40,41]. An oscillatory distance distribution between water hexamers is caused by electronic surface states [43]. Interestingly, water clusters of different apparent heights were also found along the upper edge of steps even for otherwise uncovered terraces [40,41]. The height variation suggests a variation in cluster size and/or form, and a high barrier for reorientation after water molecules adsorb to the step edge. For Pt(111) [26], similar behavior was observed.

Preferential adsorption to step edges is generally attributed to the Smoluchowski effect [44]: A smoothing of the electronic cloud at a sharp edge lowers electron density at the upper edge. This electronic redistribution was originally inferred from a dependence of the work function of metals on surface structure. However, scanning tunneling spectroscopy (STS) measurements by Avouris *et al.* visualized the local density of states (LDOS) at step edges of Au(111) and Ag(111) [45]. On the upper side, the density of unoccupied states increased at the expense of such states at the lower part of the edge. The associated dipole oriented parallel to the surface was predicted to strongly affect adsorption of molecules. Recently, different types of steps on an Ag(111) surface were also shown by STS to affect the local electronic structure in markedly different ways, indicating that all steps are not equal [46].

Water’s electron donating capacity may result in stronger binding to the upper edge. The STM results for water clusters bound to step edges on Ag(111) [40,41] and Pt(111) [26] confirm that steps are the preferred adsorption sites. They also show that diffusion is fast at the dosing temperature, and occurs over distances at least comparable to the terrace widths generally observed for a (111) plane on the time scale of the measurements. A computational study by Scipioni *et al.* finds that the adsorption energy for the water monomer to the B step type is 0.20 eV [47]. This is indeed slightly higher than the 0.18 eV found for the atop site on Ag(111) [32,48]. A comparative study of single water molecules binding to the A and B step types of Pt(111) did not find an explanation for the experimental result [49]. If the adsorption energy is in fact greater at steps, a shift to higher desorption temperatures is to be expected from surfaces with a greater step density. In so far as different step geometries also have different binding energies, surfaces with comparable densities of different step types will also have different desorption temperatures. Whether or not this is observable is a matter of experimental resolution.

In order to quantify the expected magnitude of such step-dependent TPD shifts, we suggest a simple “two-state model” to explain our results. The two states represent water molecules bound either at a terrace or a step. From all water dosed onto the surface, some fraction finds itself adsorbed as part of cyclic hexamers at (111) terrace sites, whereas the remainder is trapped at a step with a higher binding energy. The sum of these fractions equals 0.06–0.08 ML for the results shown in Figure 4. In the case of a very high step density, nearly all of the water molecules will be bound to a step. When the step density is very low—*i.e.*, close to the center of the crystal—most water will be bound in large water clusters at terraces. Furthermore, we assume that water’s binding energy for each fraction is independent of its relative occupancy. This binding energy is governed by the dissolution of the local water structure, which is expected to be different for terraces and steps. Considering our spatial resolution, on either side of the center of the crystal the mass spectrometer’s response will be a linear combination of only two contributions, *i.e.*, a contribution from the (111) terraces and a contribution from either the A or the B step type. Desorption of low coverages of water can be modeled accurately with Gaussian line shapes. The integrated signal must represent the total amount of water that was initially adsorbed and which varies only little between the various traces in Figure 4.


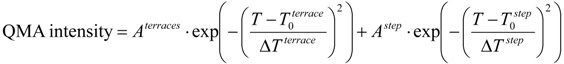
(2)


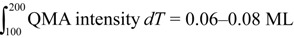
(3)

**Figure 6:**
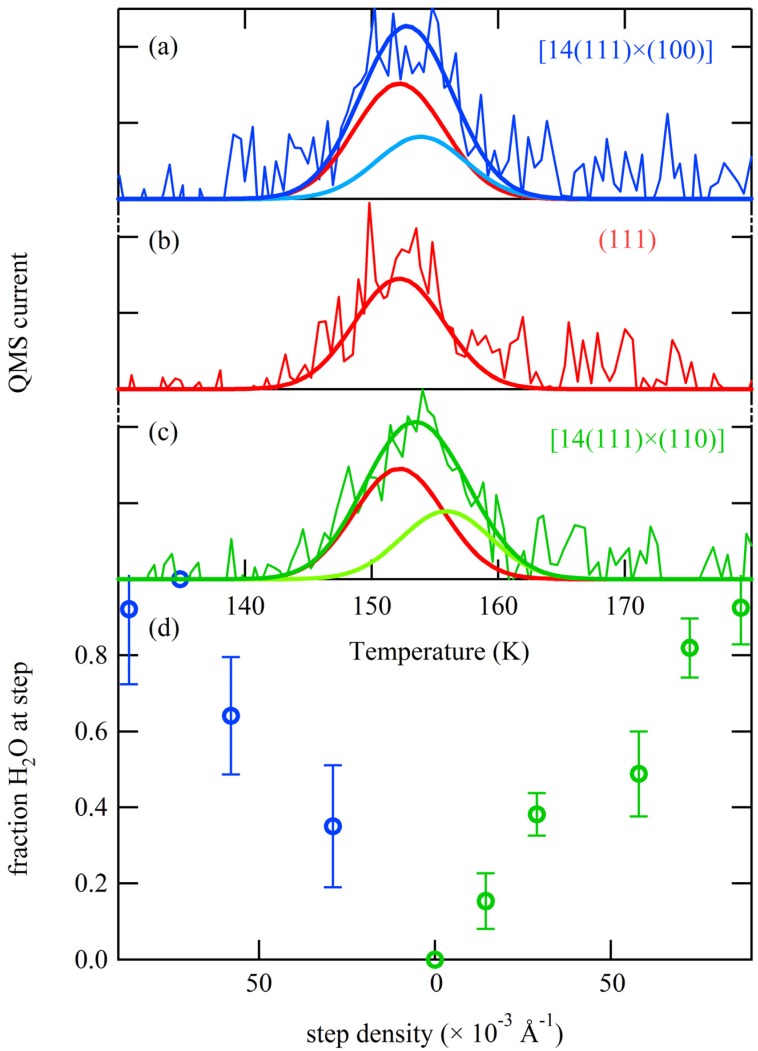
(**a**–**c**) Deconvolution of TPD features from three different surface structures into contributions from (111)-bound hexamers and water clusters bound to A (blue) and B (green) type step edges. (**d**) Fractional contribution of steps to the total observed desorption as obtained for a two-state model.

Values for *T_0_^terrace^* and *T_0_^step^* are determined from Figure 5. The near absence of steps at the central (111) part of our crystal ensures that most of the 0.06–0.08 ML H_2_O will be condensed as water hexamers, hence we use 152.2 K for *T_0_^terrace^*. To determine *T_0_^step^* for the A and B step types we take into consideration that a water hexamer spans three atomic rows. We estimate that for a 4-atom wide terrace, these hexamers cannot exist without being anchored to the step edge. Thus, we use the step density of the [4(111) × (100)] and [4(111) × (110)] planes and the linear fits in Figure 5 to estimate *T_0_^step^* for desorption from the A and B step types. These are 153.9 K and 155.9 K, respectively. Note that these values would hardly change if we would choose any other value close to N = 4. For all terrace and step desorption peaks we assume a Gaussian width (*∆T^terrace^* and *∆T^step^*) of 5.0 K, as Figure 5 suggests this to be an accurate value for both extremes. We now fit the sum of the two Gaussian contributions on either side of the middle of the crystal to our data and extract the relative amplitudes for the terrace and step contributions, *A^terrace^* and *A^step^*. The contributions by the terrace-bound hexamers (red) and steps (green and blue), as well as the total desorption intensity, are shown in Figure 6a–c for three surface structures.

The fractional contribution of the step, *i.e.*,:

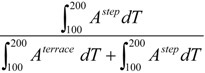
(4)
to the total desorption intensity in this two-state desorption model is shown in Figure 6d. The linearity of the step contribution on either side of (111) indicates that the model is self-consistent and fits our data well.

## 3. Experimental Section

**Figure 7:**
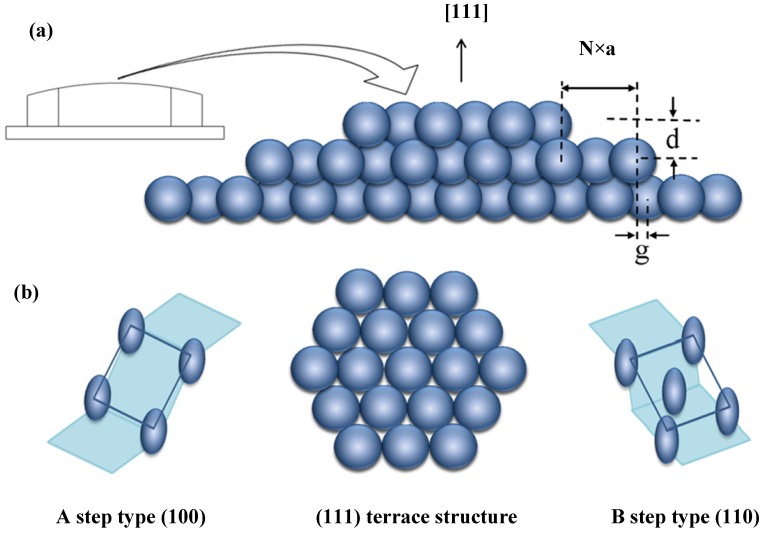
(**a**) Schematic drawing of a curved FCC metal single crystal with [111] centered at the apex; (**b**) Schematic drawing of the atomic arrangements of the A and B step types, which run parallel to the cylindrical axis (normal to the page) and separate the (111) terraces (top view).

To allow for easy variation of step type and step density, we use an Ag single crystal with a well-polished curved surface. The curvature is in principle due to atomic steps separating terraces (Figure 7a); faceting and step bunching have been observed for curved Pt [15], Au [19] and Ni [21] samples, but not Ag. Consider a cylinder of FCC crystal with its axis along the [−110] direction. Our sample is a partial slice normal to the axis, with a [111] vector radially bisecting 31° of azimuth (thus exposing Ag(111) at the apex, as in Figure 7). To one side of the apex are (100) steps (type A) running parallel to the axis, increasing in density with increasing azimuthal angle. To the other side are (110) steps (type B). Whereas the A step type consists of a square arrangement, the B step type consists of a rectangular arrangement of atoms. A Laue back reflection study indicates that our crystal’s normal is not exactly the [111] direction. It is tilted by ~0.6° in the [110] direction and ~0.3° in the [011] direction. These deviations cause the “infinite” (111) plane to be slightly off the center of the crystal’s curvature. In addition, the crystal is azimuthally rotated by ~1°. This causes a non-straight average step direction and thus a small difference in the number of R *vs.* S-type kinks.

**Figure 8:**
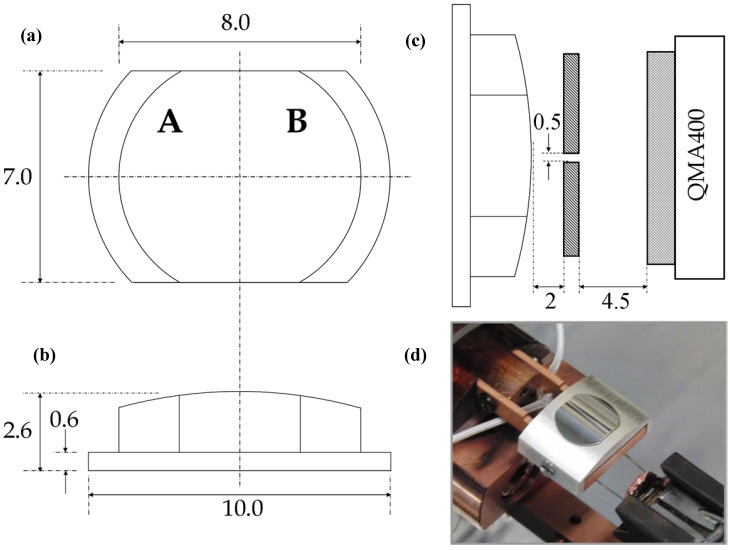
Schematic drawings (dimensions in mm) of the curved Ag crystal (**a**) front view with step type indication (**b**) bottom view (**c**) edge view of crystal in front of the differentially pumped QMS (**d**) Photograph of the crystal with retaining Ag cap.

Figure 8 shows three schematic drawings and a photograph of the actual Ag crystal used in our studies. The surface is curved and polished only on its top side. It may be considered a 31° section of a 30 mm diameter cylinder. With this angle, the surface structures range from approximately [5(111) × (100)] on the outer left side (front view) via (111) near the middle to [5(111) × (110)] on the outer right side. The initial crystal was spark eroded from an Ag boule. It was circular, 10 mm in diameter and ~ 3 mm thick. At the edge, a 1 mm wide ring was removed to leave a 0.6 mm thick flange and 8 mm diameter top surface. The sides were removed making it 7 mm wide. The curvature on the top of the crystal was initially created by spark erosion and sanding. The crystal was subsequently polished in an automated, custom built polishing machine (Surface Preparation Laboratory, Zaandam, The Netherlands).

At its 1 mm wide flange, the crystal is held by a polycrystalline Ag cap onto a Cu base plate by two screws (Figure 8d). This assembly is connected with two solid Cu leads extending from the base plate to a bath cryostat. It is electrically isolated using AlN blocks (not visible in the picture). The cryostat is inserted into the ultrahigh vacuum (UHV) chamber through an x, y, z, ϑ manipulator. Behind the base plate, a filament from a commercial light bulb (Osram, Capelle a/d IJssel, The Netherlands) enables uniform heating of the base plate and crystal. The filament is spring loaded at its glass base. Heating is performed either radiatively or by electron bombardment using a positive voltage on the crystal assembly while the filament is grounded. A type-K thermocouple is inserted into the side of the Ag polycrystalline cap. For temperature control, we use a PID controller with an internal ice point reference (Eurotherm 2416, Ashburn, VA, USA).

The crystal assembly was initially held in a home-built UHV surface science chamber for cleaning and surface structure determination using LEED. This system has a base pressure of 3 × 10^−^^10^ mbar and contains, amongst others, a sputter gun (IS 40C1, Prevac, Rogów, Poland), a QMS (Prisma 200, Pfeiffer Vacuum GmbH, Aßlar, Germany), and LEED optics (RVL2000/8/R, LK Technologies, Bloomington, IN, USA). The crystal was cleaned by sputtering-annealing cycles. We sputter using Ar^+^ (6.0 Messer, Moerdijk, The Netherlands) at 600 V and 2 μA while rotating the crystal 2° per minute, and anneal at 675 K for 10 min. LEED studies were performed with the electron beam impinging along [111] at all locations along the curved surface. The surface was translated normal to the impinging electron beam.

Subsequently, the crystal assembly was moved to a second home-built UHV chamber with a base pressure of 9 × 10^−^^11^ mbar for spatially-resolved TPD studies. Here, a Baltzers QMA400 head is kept in a differentially pumped canister that connects to the main UHV chamber via a 0.5 × 5 mm^2^ rectangular slot (Figure 8b). The curved crystal is positioned ~2 mm from this orifice for TPD studies. It is translated laterally to monitor desorption from different surface structures in separate experiments. We have modelled the geometric effects on the mass spectrometer’s instrument function for various desorption profiles. We find that, in the worst-case scenario of a broad cosine distribution, the spatial resolution is limited by the angular width of this distribution (rather than the angular width of the slit) to 3.1 mm (FWHM). Under these conditions we will detect some desorption (~10%) from the center of the crystal when performing TPD experiments at the edge of the crystal’s curvature. When desorption is more strongly directed along the local surface normal, spatial resolution increases. H_2_O (Millipore, 18.2 MΩ) was dosed onto the Ag sample at 86 K using a home-built 10 mm diameter capillary array doser at a distance large enough to ensure a uniform flux across the entire cleaned surface. The water was degassed by multiple freeze-pump-thaw cycles and backfilled with 1.1 bar He (6N, Air Products, Amsterdam, The Netherlands) prior to experiments. The H_2_O/He mixture was generally dosed onto the crystal for different durations at a fixed pressure of 1 × 10^−^^7^ mbar in the UHV chamber, as determined by an uncalibrated cold cathode gauge. Co-dosing with He allows us to dose H_2_O reproducibly, as the co-dosed He yields a large and accurately determined pressure change. Subsequent TPD experiments were performed at 1.0 K/s while monitoring *m/z* = 18. We have verified by TPD that the cold sample did not accumulate CO (*m/z* = 28) to measurable amounts prior to or during the experiment. LEED (LK Technologies RVL2000/8/R) was regularly used to verify the structure of the bare Ag surface, while AES was used to verify cleanliness (ESA 100, Staib Instruments, Langenbach, Germany).

Finally, the crystal’s surface was also studied using scanning tunnelling microscopy (STM). Here, we employ a commercial Omicron UHV system containing separate preparation and analysis chambers. The latter has a base pressure of 2 × 10^−10^ mbar and contains a variable temperature STM. We redesigned the sample holder such that the entire polished surface could be imaged, while the holder still fit inside the analysis chamber’s sample carousel. Parts of the sample holder in proximity to the sample’s polished surface were covered by an Ag foil to prevent cross-contamination of the sample during sputtering. Tungsten STM tips were prepared by electrochemical etching in NaOH using DC current. Under UHV conditions, tip treatment also included heating the tip apex with a 100–500 μA electron emission current to remove tungsten oxide and tip stabilisation by applying 2–3 V pulses wile scanning. We used AES (VG 100AX hemispherical analyzer in combination with a LEG-63 electron gun) and LEED (VG RVL900) regularly to verify cleanliness and long-range surface order. AES spectra never showed any sign of adsorbed oxygen, O_ads_, on any of the stepped (111) surfaces, even after prolonged exposure (~10^3^ mbar × s) to O_2_ at room temperature.

## 4. Conclusions

We have presented a study of water desorption from a curved single crystal surface. We find a shift in the desorption temperature for very small coverages that we believe originates from small differences in the dissolution and consecutive desorption energies of water clusters bound to the (111) terrace and the upper edge of steps. The stronger shift for the B step type indicates a stronger binding to this (110) edge. We note that the same trend has been observed on Pt, where too the B type step induces a larger temperature shift than the A step type in desorption of the last water molecules [28]. This observation may hint at a more general rule regarding the bond strength of water to step edges, and a relation to the size of the Smolukowski effect at A and B step types for FCC metals.

Our study furthermore demonstrates major experimental advantages of using curved samples in surface science: In addition to the time saved by not swapping crystals, the random temperature-measurement error introduced by sample-to-sample variations in thermal contact is eliminated. With demonstrably low scatter of the temperature readings, we can find evidence for a very small difference in binding energy between water clusters at (111) terrace sites and the A and B step types on this noble metal. It is unlikely that such sensitivity could be achieved with a conventional study of multiple flat single crystals. Thus, our approach makes finer quantitative distinctions, which can in turn serve as benchmarks for theoretical calculation of binding energy differences between closely related adsorption sites.
